# Prevalence of Illness Anxiety Disorder among Health Colleges Students in King Saud University, Riyadh, Saudi Arabia

**DOI:** 10.1192/j.eurpsy.2025.1006

**Published:** 2025-08-26

**Authors:** A. N. Alhadi, R. B. Almashaan, R. M. Alshehri, R. A. Alhazmi, N. A. Alfawaz, M. M. Alsuliman, A. A. Almarzuqi

**Affiliations:** 1 Depatment of Psychiatry, College of Medicine; 2 College of Medicine, King Saud University, Riyadh, Saudi Arabia

## Abstract

**Introduction:**

Illness Anxiety Disorder denotes an excessive fear of becoming critically ill. Even when a medical exam does not indicate a significant medical problem, people may have no physical symptoms or think that familiar body sensations or minor symptoms are symptoms of a dangerous sickness.

**Objectives:**

We aimed to estimate and compare the prevalence of illness anxiety disorder among health colleges students at King Saud University. Also, we examined whether the prevalence of illness anxiety disorder is influenced by the specific field of study within the medical disciplines and whether the disorder manifests differently among students based on their chosen area of specialization.

**Methods:**

This was an observational cross-sectional study. Data were collected through an online self-administered questionnaire called The Short Health Anxiety Inventory (SHAI) and was analyzed using SPSS 23rd version.

**Results:**

The prevalence of IAD in this study was found to be 5.8% (n=325). with a significant relation to gender, college, the presence of psychiatric disorders, and being subjected to abuse or neglect.

**Conclusions:**

This study found that the prevalence of illness anxiety disorder was low compared to other studies published in the past.

**Disclosure of Interest:**

None Declared

